# ADAM9 functions as a transcriptional regulator to drive angiogenesis in esophageal squamous cell carcinoma

**DOI:** 10.7150/ijbs.65488

**Published:** 2021-09-07

**Authors:** Yu-Sen Lin, Ting-Ting Kuo, Chia-Chien Lo, Wei-Chung Cheng, Wei-Chao Chang, Guan-Chin Tseng, Shih-Ting Bai, Yu-Kai Huang, Chih-Ying Hsieh, Han-Shui Hsu, Yi-Fan Jiang, Chen-Yuan Lin, Liang-Chuan Lai, Xing-Guo Li, Yuh-Pyng Sher

**Affiliations:** 1 Graduate Institute of Clinical Medical Science, China Medical University, Taichung 404, Taiwan.; 2 Division of Thoracic Surgery, China Medical University Hospital, Taichung 404, Taiwan.; 3 Center for Molecular Medicine, China Medical University Hospital, Taichung 404, Taiwan.; 4 Graduate Institute of Biomedical Sciences, China Medical University, Taichung 404, Taiwan.; 5 Department of Anatomic Pathology, Nantou Hospital of the Ministry of Health and Welfare, Nantou 540, Taiwan.; 6 Division of Thoracic Surgery, Department of Surgery, Taipei Veterans General Hospital, Taipei 112, Taiwan.; 7 Institute of Emergency and Care Medicine, School of Medicine, National Yang-Ming University, Taipei 112, Taiwan.; 8 Graduate Institute of Molecular and Comparative Pathobiology, School of Veterinary Medicine, National Taiwan University, Taipei 106, Taiwan.; 9 School of Pharmacy, China Medical University, Taichung 404, Taiwan.; 10 Division of Hematology and Oncology, China Medical University Hospital, Taichung 404, Taiwan.; 11 Graduate Institute of Physiology, College of Medicine, National Taiwan University, Taipei 100, Taiwan.; 12 Chinese Medicine Research Center, China Medical University, Taichung 404, Taiwan.

**Keywords:** ESCC, ADAM9, PAI-1, angiogenesis, transcriptional regulator

## Abstract

Hypoxia and angiogenesis play key roles in the pathogenesis of esophageal squamous cell carcinoma (ESCC), but regulators linking these two pathways to drive tumor progression remain elusive. Here we provide evidence of ADAM9's novel function in ESCC progression. Increasing expression of ADAM9 was correlated with poor clinical outcomes in ESCC patients. Suppression of ADAM9 function diminished ESCC cell migration and *in vivo* metastasis in ESCC xenograft mouse models. Using cellular fractionation and imaging, we found a fraction of ADAM9 was present in the nucleus and was uniquely associated with gene loci known to be linked to the angiogenesis pathway demonstrated by genome-wide ChIP-seq. Mechanistically, nuclear ADAM9, triggered by hypoxia-induced translocation, functions as a transcriptional repressor by binding to promoters of genes involved in the negative regulation of angiogenesis, and thereby promotes tumor angiogenesis in plasminogen/plasmin pathway. Moreover, ADAM9 suppresses plasminogen activator inhibitor-1 gene transcription by interacting with its transcription factors at the promoter. Our findings uncover a novel regulatory mechanism of ADAM9 as a transcriptional regulator in angiogenesis and highlight ADAM9 as a promising therapeutic target for ESCC treatment.

## Introduction

Esophageal cancer is one of the most fatal types of malignancies, with rapid tumor mass growth and frequently a poor prognosis 1. The histological type of this disease is disproportionate between Asian and Caucasian patients 2. In contrast to Western countries where esophageal adenocarcinoma predominates, esophageal squamous cell carcinoma (ESCC) occurs more commonly in Asian populations, with, for example, a ratio of 10-15:1 squamous cell carcinoma to adenocarcinoma in Taiwan 3. The 5-year survival of ESCC is rarely more than 25% even after curative-intent resection 4. More than 50% of tumors recur within one year after surgery due to locoregional recurrence and distal metastases 5. The molecular mechanism associated with ESCC progression is unclear and limits improvements in the clinical therapy of ESCC patients.

Hypoxia-associated biomarkers are associated with poor outcomes in esophageal cancer 6. Under hypoxic conditions, angiogenic factors are induced that promote tumor angiogenesis, which is a critical factor for the progression of solid tumors 7. The plasminogen activating system can increase local proteolysis for a proangiogenic effect, thereby promoting tumorigenesis. For example, tissue plasminogen activator (tPA) is required for the growth, invasion, and angiogenesis of pancreatic cancer, and urokinase plasminogen activator (uPA) is a strong prognostic factor in adenocarcinoma of the esophagus 8, 9. Plasminogen activator inhibitor type 1 (PAI-1) can bind to plasminogen activators (tPA and uPA) to influence the plasminogen activator pathway and prevent the generation of plasmin 10. Based on this, PAI-1 was considered to have antitumorigenic effects. However, a paradoxical association between PAI-1 and an unfavorable clinical outcome was reported, where PAI-1-mediated tumor angiogenesis is considered regardless of angiogenic stimuli in malignant pleural mesothelioma 11. Although PAI-1 has been reported as a potential marker for the malignancy of ESCC 12, the role of PAI-1 in angiogenesis relating to plasminogen activator-based pathways in ESCC remains unclear.

Tumor metastasis involves degradation of the extracellular matrix and requires several proteolytic enzymes, such as matrix metalloproteinases, and a disintegrin and metalloproteases (ADAMs) either from tumor cells or surrounding stromal cells in ESCC 13, 14. ADAM9, a type I transmembrane protein of the ADAM family, is involved in cell adhesion and migration via its disintegrin domain for adhesion with integrin and its metalloproteinase domain for ectodomain shedding to release a number of molecules with important roles in tumorigenesis and angiogenesis 15. ADAM9 is linked with tumor progression in different types of cancer. For example, ADAM9 promotes lung cancer metastasis to brain by a plasminogen activator-based pathway and increases angiogenesis 16, 17. In ESCC, up-regulated DNA methyltransferase 1 contributes to tumor growth through ADAM9-mediated epidermal growth factor receptor (EGFR)-AKT signaling 18. However, the role of ADAM9 in ESCC tumorigenesis has not been fully elucidated. In this study, we showed that ADAM9 enhances the plasminogen activator-based pathway for ESCC progression. Notably, ADAM9 can translocate to the nucleus and occupy chromatin for the inhibition of transcription of genes in negative regulation of angiogenesis such as PAI-1 and PGK1, thereby promoting angiogenesis in ESCC.

## Materials and Methods

### Patient information

Formalin-fixed paraffin-embedded ESCC samples were obtained from 213 patients who received curative esophagectomy between August 2000 and September 2009 at China Medical University Hospital in compliance with protocols approved by the hospital's institutional review board (DMR98-IRB-059). The informed consent was obtained for experimentation with human subjects. Clinical staging and clinicopathological TNM classification were determined according to criteria proposed by the American Joint Committee on Cancer, 6^th^ edition.

### ESCC tumor animal models

SCID mice (8 weeks of age) were used as models of ESCC distal metastasis by intracardial injection with control and *ADAM9* knockdown CE146T or KYSE170 cells (5×10^4^). All experimental procedures were approved by the Institutional Animal Care and Use Committee of China Medical University and Hospital. After two months, the mice were sacrificed and tumors were excised and weighed.

### Transmission electron microscopy

For immunogold EM analysis of ADAM9, samples were performed as previously described 19. Briefly, the ESCC cells were fixed by 4% paraformaldehyde and 0.25% glutaraldehyde in 0.1 M phosphate buffer (pH 7.3). After rinsing with 0.1 M phosphate buffer (pH 7.3), the cell pellets were post-fixed in 4% low melting agar. The agar samples were dehydrated with ascending concentrations of ethanol, then infiltrated and embedded with Spurr's resin at 65 degrees for 16 hours. 100-nm-thick sections were stained with anti-ADAM9 antibody (AF939, R&D, 1:500) and 12-nm-gold anti-goat antibody (1:20). The stained specimens were imaged with the FEI Tecnai G2 TF20 Super TWIN microscope operating at 120 kV.

### Immunofluorescence and Proximity Ligation Assay

The experiments were conducted as previously described 20. Briefly, for immunofluorescence, cells were cultured for 24 h on coverslips, fixed with 4% paraformaldehyde pH 7.4 in PBS for 15 min at room temperature, and blocked with 0.1% tween-PBS and 5% BSA for 1 h at 37 °C. Incubations with primary antibodies were performed overnight at 4 °C. After incubation with secondary antibodies for 1 h at 37 °C, cells were stained with DAPI (4',6'-diamidino-2-phénylindole) and mounted using Vectashield Hardset mounting medium (Vector Laboratories, H-1400). PLA assays were conducted using Duolink™ *In situ* Red Starter Kit (#DUO92101, Sigma-Aldrich) according to the recommendations. The primary antibodies against ADAM9 (AF939; R&D), TP53 (#2524; CST), HIF1α (#610959; BD), and USF1 (sc-390027; santa cruz) were used.

### Statistical analysis

Comparisons between the analyzed parameters were performed using Student's t-test or two-way ANOVA for continuous variables. All statistical tests were two-sided. Survival curves were obtained by the Kaplan-Meier method. Statistical significance was set for all tests at P < 0.05.

## Results

### ADAM9 overexpression is associated with poor outcomes of ESCC patients

**Figure 1:**
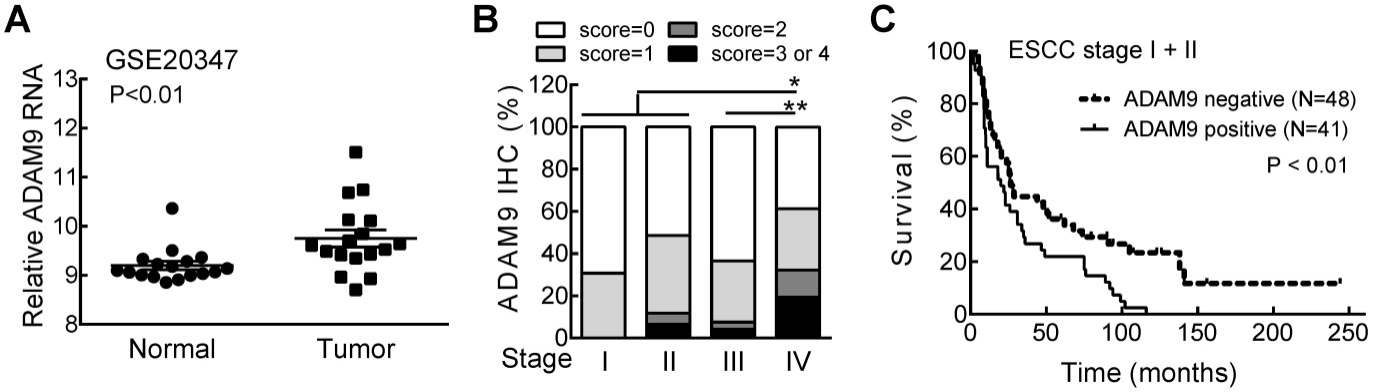
** ADAM9 is overexpressed in patients with advanced ESCC and correlated with poor outcome. (A)** The RNA levels of ADAM9 in the specimens from ESCC patients in the GSE20347 dataset. N = 17, each group. **(B)** The distribution of ADAM9 IHC scores in tumor specimens from 4 stages of ESCC. Wilcoxon rank sum test. *, P < 0.05; **, P < 0.01. **(C)** Kaplan-Meier survival curve of ESCC patients with stage I and II grouped by ADAM9 IHC staining status (positive or negative).

We first investigated the relationship of ADAM9 with the clinicopathological parameters of ESCC patients, and found significantly higher levels of ADAM9 mRNA in ESCC tumors than in normal esophageal tissues from the Gene Expression Omnibus (GEO) databases (GSE20347) (Figure 1A). Next, we examined the ADAM9 proteins in paraffin-embedded ESCC specimens by immunohistochemical (IHC) staining with anti-ADAM9 antibody 16. Different staining levels of ADAM9 in ESCC tissues were scored from 0 to 4 by a pathologist, and 44% of 213 specimens were scored greater than 0 as positive ADAM9 staining (Table S1). In stage IV patients, the percentage of positive ADAM9 staining (61%) and the staining intensity represented by the IHC score were significantly higher than in all other stages (Figure 1B). Among early stage ESCC patients (stage I and II), those with positive ADAM9 staining had a shorter survival time compared with the ADAM9-negative group (Figure 1C), but that trend was not observed in late stages (stage III and IV, Figure S1), probably due to the very short survival time of late stage-patients. These data reveal that ADAM9 is correlated with tumor progression in ESCC.

### ADAM9 suppression reduces the migration of ESCC cells and metastasis of ESCC tumor xenografts

**Figure 2:**
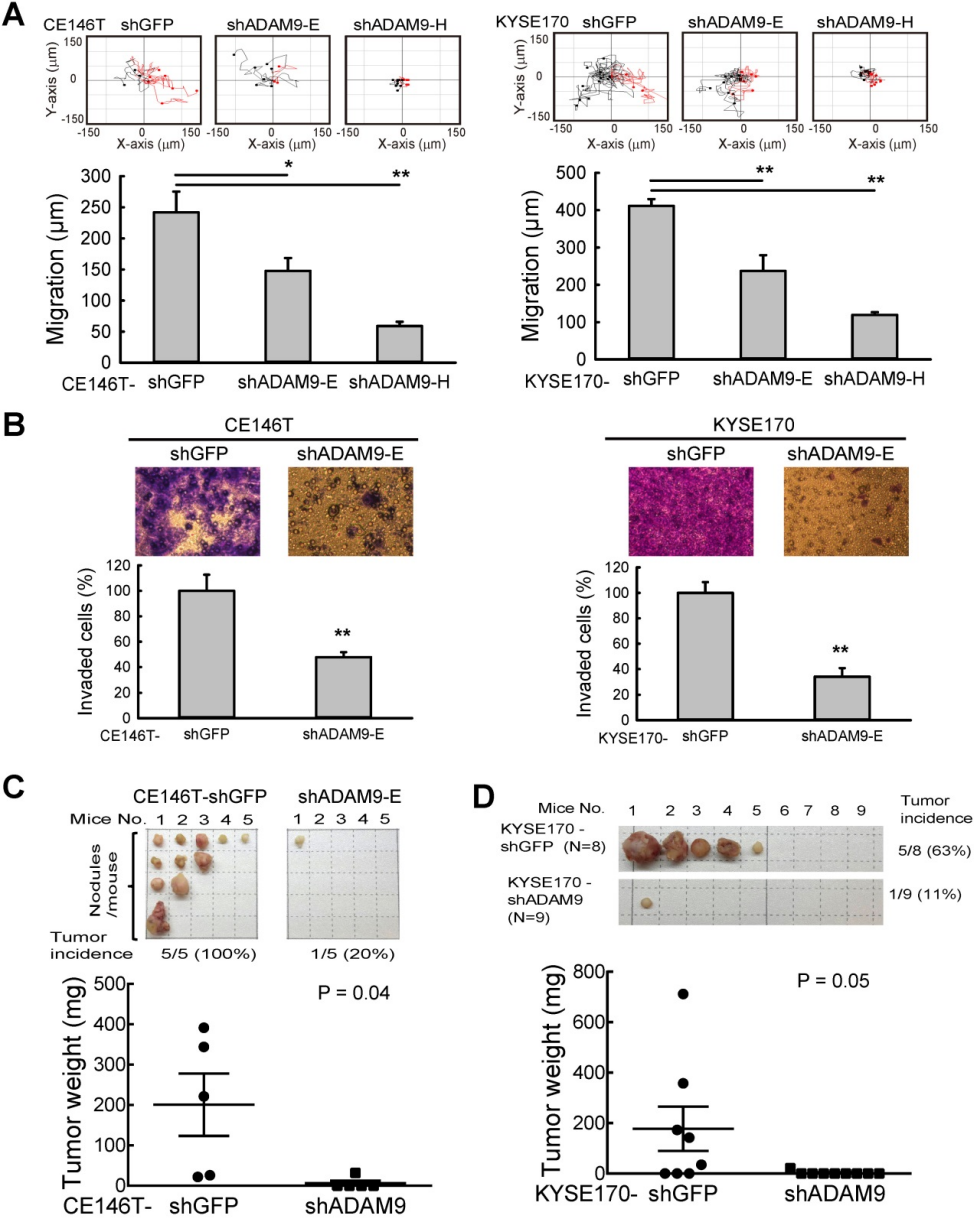
***ADAM9* knockdown reduces ESCC cell growth and metastasis in animal models. (A)** Videomicroscopy of individual cells is quantitated to determine the distance and direction of cellular migration of CE146T and KYSE170 cells. Black indicates migration to the left and red indicates migration to the right. Bar chart data are mean ± SD. **(B)** Crystal violet staining of control and *ADAM9* knockdown cells in the transwell assays that migrated with Matrigel after 2 days. One of three independent assays performed in triplicate is shown. **(C)** Control or *ADAM9* knockdown CE146T cells were inoculated into the left cardiac ventricle of 6~8-week-old SCID mice (N=5 in each group). The mice were sacrificed on day 64. The metastatic nodules were collected from the whole body of each mouse and weighed for analysis. **(D)** Intracardiac injection of control or *ADAM9* knockdown KYSE170 cells into 10-week-old SCID mice (N=8 in each group). The mice were sacrificed on day 48. Metastatic tumors were weighed for analysis. Student's *t* test. *, P < 0.05. **, P < 0.01.

To investigate the role of ADAM9 in ESCC progression, *ADAM9* knockdown cells were generated using lentiviral shRNAs targeting different regions of *ADAM9*. No obvious change in cell proliferation rate was detected in control versus *ADAM9* knockdown cells by MTT assays over a period of 3 days (Figure S2A-B). However, we found that control cells migrated longer distances than *ADAM9* knockdown cells when tracking each cell's movement with time-lapse detection (Figure 2A and Figure S2C). Moreover, compared to control cells, *ADAM9* knockdown ESCC cells significantly decreased the invasion ability in a transwell assay (Figure 2B and Figure S2D). Thus, ADAM9 promotes the invasion and migration ability of ESCC cells.

To further examine the metastatic effect of ADAM9, control and *ADAM9* knockdown CE146T cells were intracardially injected into SCID mice for evaluating distal metastasis. In the control group, in which mice were injected with the control CE146T cells, all mice (100%) developed several distal metastatic tumors within 64 days after cancer cell injection, whereas only one mouse (20%) had one small distal metastatic tumor in the *ADAM9* knockdown group (Figure 2C). The metastatic nodules in the control group grew in the neck, ribs, kidney, and subcutaneous regions. Similar results were observed in mice injected with control and *ADAM9* knockdown KYSE170 cells (Figure 2D). Although only one distant metastatic tumor was found in each mouse, the tumor incidence was 63% (5 of 8) in the control group, markedly higher than 11% (1 of 9) in the *ADAM9* knockdown group. Notably, the tumor size was significantly bigger in the control group than in the *ADAM9* knockdown group. These data indicate that ADAM9 promotes tumor metastases in ESCC.

### ADAM9 regulates plasminogen activator-based pathway in ESCCs

**Figure 3:**
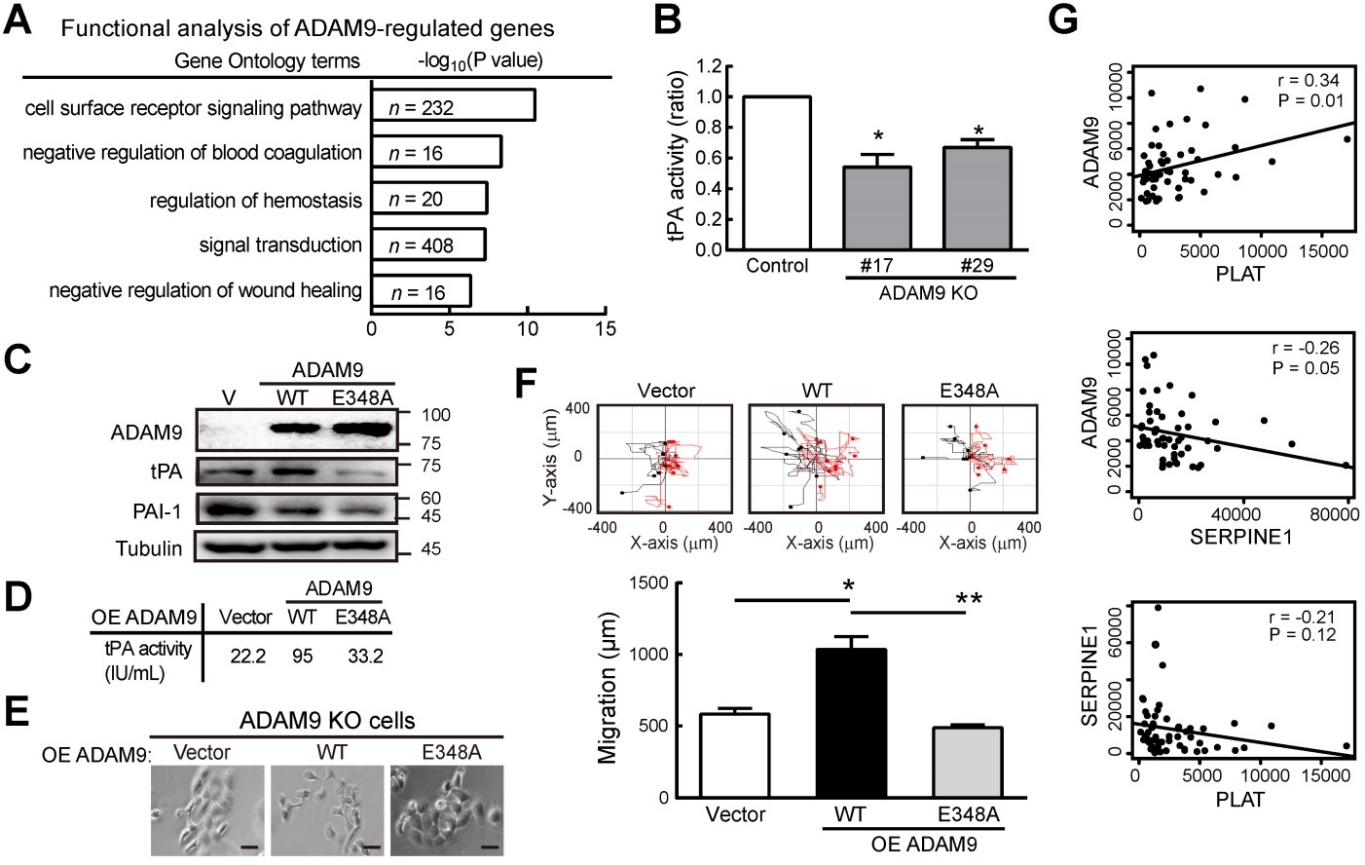
**ADAM9 promotes the plasminogen activator-based pathway in ESCC. (A)** Gene ontology-based functional analysis of ADAM9-regulated genes using differentially expressed genes in the control and ADAM9 KO KYSE170 cells. **(B)** tPA activity measured in the two-day culture conditioned media. Two individual clones of ADAM9 KO (#17 and #29) KYSE170 cells were analyzed. **(C)** Western blot analysis of indicated proteins in *ADAM9* KO KYSE170 cells transiently transfected with plasmids of vector (V), ADAM9 wild type (WT), and E348A mutant. **(D - F)** The measured tPA activity (**D**), cell morphology; scale bar, 200 μm (**E**), and migration distance (**F**) of *ADAM9* KO cells transiently transfected with plasmids of ADAM9 WT and E348A mutant. Bar chart data are mean ± SD. Student's *t* test. *, P < 0.05. **, P < 0.01.** (G)** The correlation of *ADAM9* and *PLAT* or *SERPINE1* RNA expression in ESCC patients from TCGA dataset.

To further investigate how ADAM9 can promote the pathogenesis of ESCC, we generated stable cell clones using a CRISPR/Cas9 system to knock out (KO) *ADAM9* and demonstrated that cell proliferation was reduced in *ADAM9* KO KYSE170 cells (Figure S3A). The reduced cell proliferation in *ADAM9* KO cells but not in *ADAM9* KD cells is likely due to the complete reduction of ADAM9 expression in KO cells. Next, we explored ADAM9-regulated genes by comparing transcriptomes between control and *ADAM9* KO cells. Notably, we found genes in the plasminogen activator system (*PLAT* and *SERPINE1*) in the top three functional enrichment analysis (Figure 3A). Given that the plasminogen activation system plays a central role in the regulation of a variety of pathological processes including malignancy, chemoresistance, coagulation, and angiogenesis 21, 22, we further examined whether ADAM9 regulated the plasminogen activation system for the pathogenesis of ESCC cells.

First, we demonstrated a decreased tPA activity in *ADAM9* KO KYSE170 cells compared to control cells (Figure 3B). Accordingly, we found decreased RNA levels of tPA (encoded by *PLAT*) but increased PAI-1 (encoded by *SERPINE1*) in *ADAM9* knockdown cells compared with control cells (Figure S3B). Moreover, the secretion of tPA proteins into the culture media was also significantly decreased in *ADAM9* knockdown TE and KYSE170 cells (Figure S3C). To further investigate whether the plasminogen activator-based pathway was regulated by the protease activity of ADAM9 in ESCC, we introduced ectopic ADAM9 wild-type (WT) or catalytic mutant E348A 23, which is defective in the protease activity, into *ADAM9* KO cells. Compared to WT, the ADAM9 protease mutant E348A decreased tPA expression (Figure 3C) and reduced the tPA activity by nearly 3-fold (Figure 3D). In terms of cell morphology and motility, we found that overexpression of ADAM9 WT showed more cell scattering, a dispersion of compact cells (Figure 3E), and increased cell migration ability (Figure 3F), whereas expression of the E348A mutant in *ADAM9* KO cells retained the cell contact and migration characteristics of vector control cells.

To investigate whether the role of ADAM9 for promoting the plasminogen activator-based pathway is clinically relevant, we measured the correlation of *ADAM9* and *PLAT* or *SERPINE1* expression in ESCC specimens from The Cancer Genome Atlas (TCGA) dataset. In patients with high ADAM9 expression (above the mean), a positive correlation of *ADAM9* and *PLAT* RNA levels was detected (r = 0.34), whereas a negative correlation of *ADAM9* and *SERPINE1* levels was detected (r = - 0.26) (Figure 3G). Notably, we found a poor correlation of *PLAT* and *SERPINE1* RNA (r = -0.21; P = 0.12), suggesting that the correlation of *ADAM9* and *PLAT* or *SERPINE1* was not caused by a negative correlation between *PLAT* and *SERPINE1* (Figure 3G). Taken together, these results show that ADAM9 can promote the pathogenesis of ESCC by activating the plasminogen activator-based pathway.

### Nuclear ADAM9 is increased in ESCC under hypoxia

**Figure 4:**
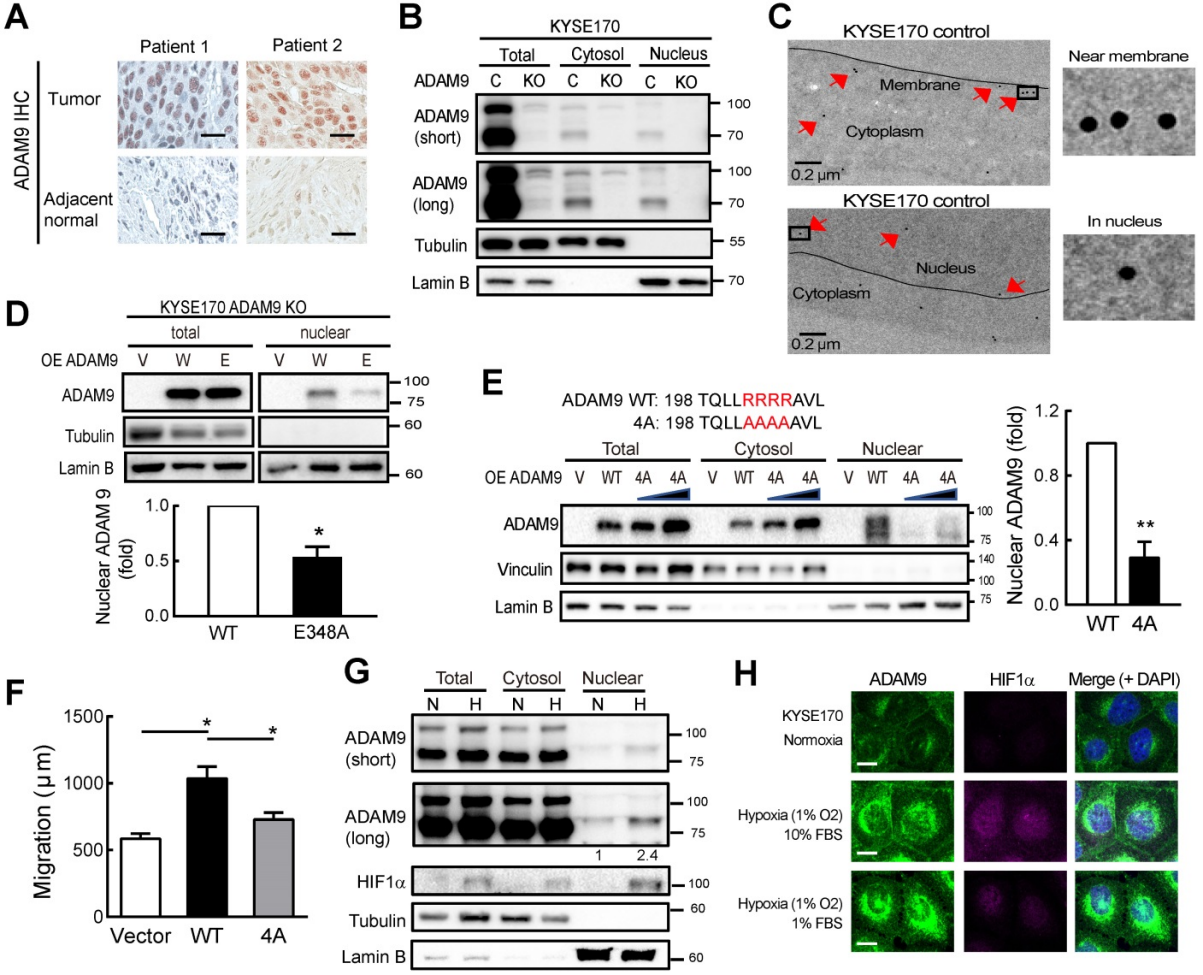
**ADAM9 protein is detected in the nuclei of ESCC cells. (A)** Representative image of ADAM9 IHC staining in tumor and adjacent normal tissue in ESCC specimens. Scale bar, 25 μm. **(B)** Nuclear localization of ADAM9 in control and ADAM9 KO KYSE170 cells. C, control; KO, ADAM9 KO. Short or long, short or long exposure. **(C)** Presence of ADAM9 in cell membrane and nucleus of KYSE170 cells using transmission electron microscopy. ADAM9 is detected using 15 nm gold particles. Arrows indicate the positive signals for ADAM9 detection (top). The highlighted square areas are shown (right). **(D)** Cellular fractionation followed by western blot analysis in *ADAM9* KO KYSE170 cells transiently transfected with plasmids of ADAM9. W, wild type and E, E348A mutant. Representative images of total proteins and nuclear proteins using the same antibody staining from the same blots. Nuclear ADAM9 protein levels were calculated from three independent assays. **(E)** Western blot analysis in *ADAM9* KO KYSE170 cells transiently transfected with plasmids of ADAM9 WT and 4A mutant. One and two-fold of plasmid amount of ADAM9 4A mutant were used. Nuclear ADAM9 protein levels were calculated from three independent assays. **(F)** Migration ability of *ADAM9* KO KYSE170 cells transiently transfected with plasmids of ADAM9 WT and 4A mutant. **(G)** Western blot analysis of KYSE170 cells treated with normoxic or hypoxic conditions. The amount of ADAM9 protein relative to the normoxia group was shown. **(H)** Fluorescence immunohistochemistry of ADAM9 and HIF1α in KYSE170 cells with confocal microscopy. Hypoxia, 1% O_2_. Scale bars, 20 µm.

IHC staining of ESCC specimens demonstrated that ADAM9 was more highly expressed in esophageal tumor cells, as compared to adjacent normal esophageal tissue (Figure 4A). Notably, ADAM9-positive staining was clearly detected in the nuclei of ESCC specimens despite that ADAM9 is a membrane protein. To validate this observation, we performed cellular fractionation and showed that ADAM9 proteins were indeed detected in the nuclear fraction of ESCC cells, using two different antibodies against the N-terminal and C-terminal domains of ADAM9, the signals of which were markedly suppressed in *ADAM9* knockdown cells (Figure S4A). Similar results were also observed in control and *ADAM9* KO KYSE170 cells (Figure 4B). Furthermore, we performed transmission electron microscopy (TEM) with immunogold labeling and validated the localization of ADAM9 in the membrane and nuclei of KYSE170 cells (Figure 4C) whereas no signals were detected in ADAM9 KO cells (Figure S4B). Notably, ADAM9 E348A protease mutant showed much lower nuclear ADAM9 levels compared to WT by Western blot (Figure 4D) and confocal microscopy (Figure S4C).

NLS (nuclear localization signal) is known to contain three to five basic residues that are required for nuclear translocation and interaction with importin β1. Using two independent prediction programs, cNLS Mapper program 24 and NucPred program 25, we found one putative NLS containing 4 arginine residues R202 to R205 adjacent to metalloprotease domain in the N-terminal of ADAM9. By replacing 4 arginine sites to alanine in ADAM9 NLS (4A), the nuclear translocation of ADAM9 was decreased compared with ADAM9 WT (Figure 4E). Moreover, the migration ability was also disrupted in cells expressing with ADAM9 4A compared with ADAM9 WT (Figure 4F).

A high level of hypoxia inducible factor 1 subunit alpha (HIF1α was detected in the esophageal tissue of ESCC patients and correlated with clinical TNM stage and poor outcomes 26. We then investigated whether hypoxia could induce nuclear translocation of ADAM9 in ESCC. Cellular fractionation revealed that nuclear ADAM9 was increased under hypoxic conditions (Figure 4G) or induced by CoCl_2_ treatment in a time-dependent manner (Figure S4D). Moreover, as shown by confocal fluorescence, co-localization of ADAM9 with DAPI (4′,6-diamidino-2-phenylindole) in the nucleus was increased by hypoxia (achieved by culturing in 1% O_2_), as well as under stressed conditions of low serum with hypoxia (Figure 4H). Taken together, ADAM9 proteins can translocate to the nucleus of ESCC cells, especially under hypoxia.

### Nuclear ADAM9 regulates genes involved in angiogenesis in ESCC

**Figure 5:**
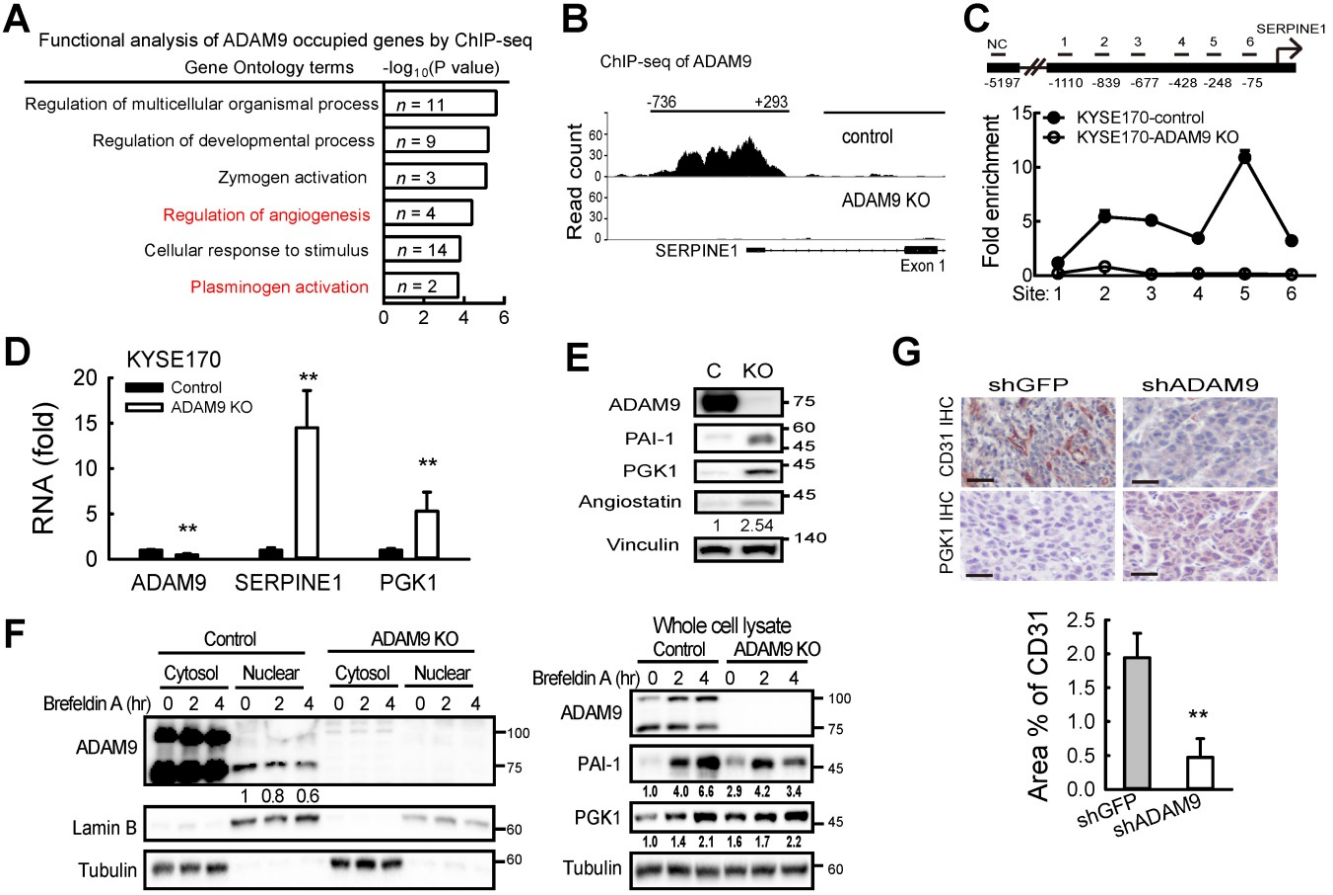
** Nuclear ADAM9 protein occupies DNA and regulates gene expression to promote angiogenesis in ESCC cells. (A)** Gene ontology-based functional analysis of target genes occupied by ADAM9. **(B)** Peaks of ADAM9 occupancy were identified in the *SERPINE1* promoter by ADAM9 ChIP-sequencing in KYSE170 control and *ADAM9* KO cells. The *SERPINE1* gene location is shown at the bottom. **(C)** ChIP-qPCR enrichment analysis with primer sets (1 to 6) in the *SERPINE1* promoter region in control and *ADAM9* KO KYSE170 cells using ADAM9 antibody and control IgG. Data are mean ± SD. **(D)** RT-qPCR of *ADAM9*, *SERPINE1*, and *PGK1* in control and ADAM9 knockout KYSE170 cells. **(E)** Western blot analysis of angiostatin proteins in control and *ADAM9* KO KYSE170 cells. Cells were cultured in starvation for 3 days. **(F)** Cellular fractionation followed by western blot analysis of control and *ADAM9* KO KYSE170 cells treated with 5 μM Brefeldin A at different time points. **(G)** Representative images of tumor tissues processed for IHC staining of PGK1 and CD31 from metastatic KYSE170 tumor animal models. Scale bar, 50 μm. CD31 staining was quantified as the percentage of positively stained area (bottom). Bar chart data are mean ± SD. Student's *t* test. *, P < 0.05. **, P < 0.01.

Next, to investigate whether nuclear ADAM9 can bind to DNA for genetic regulation, we performed chromatin immunoprecipitation (ChIP) using an antibody that can pull down ADAM9 in ESCC cells (Figure S5A). A genome-wide survey by ChIP-seq was carried out to determine the binding profile of nuclear ADAM9 by comparing control and *ADAM9* KO KYSE170 cells. The top 10 specific peaks of ChIP-seq were identified (Table S2). Gene ontology-based functional analysis reveal that these top hits are involved in functions, such as regulation of angiogenesis and plasminogen activation (Figure 5A). Notably, *SERPINE1* gene (encoding PAI-1) is listed in all these functions (Figure 5A). By deep analysis of the binding regions from the ChIP-seq profile, ADAM9 occupied the *SERPINE1* promoter from position -736 to 293, with a width of 1030 bp in control cells but no signals in *ADAM9* KO KYSE170 cells (Figure 5B). ADAM9's occupation of the promoter of *SERPINE1* was confirmed using ChIP-quantitative PCR (ChIP-qPCR), which showed enrichment over an extended region (from site 2 to 6) in control compared to *ADAM9* KO KYSE170 cells (Figure 5C). Similar results were observed in control (shGFP) TE cells compared with *ADAM9* knockdown TE cells (Figure S5B).

Of the two genes, *SERPINE1* and *PGK1* (encoding phosphoglycerate kinase 1), identified for their roles in regulating plasminogen activation (Figure 5A), we found that ADAM9 also occupied the *PGK1* promoter (Figure S5C). PGK1 has been reported to act as a disulfide reductase to stimulate proangiogenic plasmin to release the blood vessel inhibitor angiostatin, which blocks angiogenesis 27. Importantly, we found that the levels of *SERPINE1* and *PGK1* mRNAs were increased in *ADAM9* KO and knockdown cells (Figure 5D and Figure S5D), as well as increased protein levels of PAI-1, PGK1, and angiostatin in *ADAM9* KO cells compared with control cells (Figure 5E). Conversely, ectopic expression of ADAM9 decreased PGK1 expression in KYSE170-ADAM9 KO cells (Figure S5E).

Next, we asked whether suppression of ADAM9 nuclear translocation affects PAI-1 and PGK1 expression. To this end, we treated control and ADAM9 KO KYSE170 cells with brefeldin A (BFA), an inhibitor of intracellular protein transport. The levels of nuclear ADAM9 were decreased and the total PAI-1 and PGK1 proteins were increased in the control cells in a time-dependent manner (Figure 5F). In ADAM9 KO cells, PAI-1 initially increased after 2 h of BFA treatment but then declined to a level equivalent to that at the zero timepoint whereas PGK1 levels remained constant (Figure 5F). We speculated that the negative association between the amounts of nuclear ADAM9 and the PAI-1 or PGK1 proteins may be due to the transcriptional repression by nuclear ADAM9.

Next, to validate ADAM9 function in angiogenesis, we demonstrated that endothelial marker CD31 was higher in control tumors than in *ADAM9* knockdown KYSE170 tumors whereas IHC staining of PGK1 was lower in control tumors than *ADAM9* knockdown KYSE170 tumors (Figure 5G). Moreover, angiogenesis (tube formation) was reduced in human umbilical vein endothelial cells (HUVECs) cultured in conditioned media from *ADAM9* KO cells (Figure S5F). Conditioned media from *ADAM9* KO cells with overexpression of ADAM9 WT strongly restored angiogenesis in HUVECs whereas conditioned media from *ADAM9* KO cells with overexpression of the E348A mutant only slightly restored angiogenesis (Figure S5G). Taken together, these results reveal that nuclear ADAM9 functions as a transcriptional repressor by binding to promoters of genes involved in the negative regulation of angiogenesis, and thereby promotes tumor angiogenesis.

### ADAM9 suppresses *SERPINE1* transcription by interacting with known transcription factors for *SERPINE1* gene regulation in ESCC

**Figure 6:**
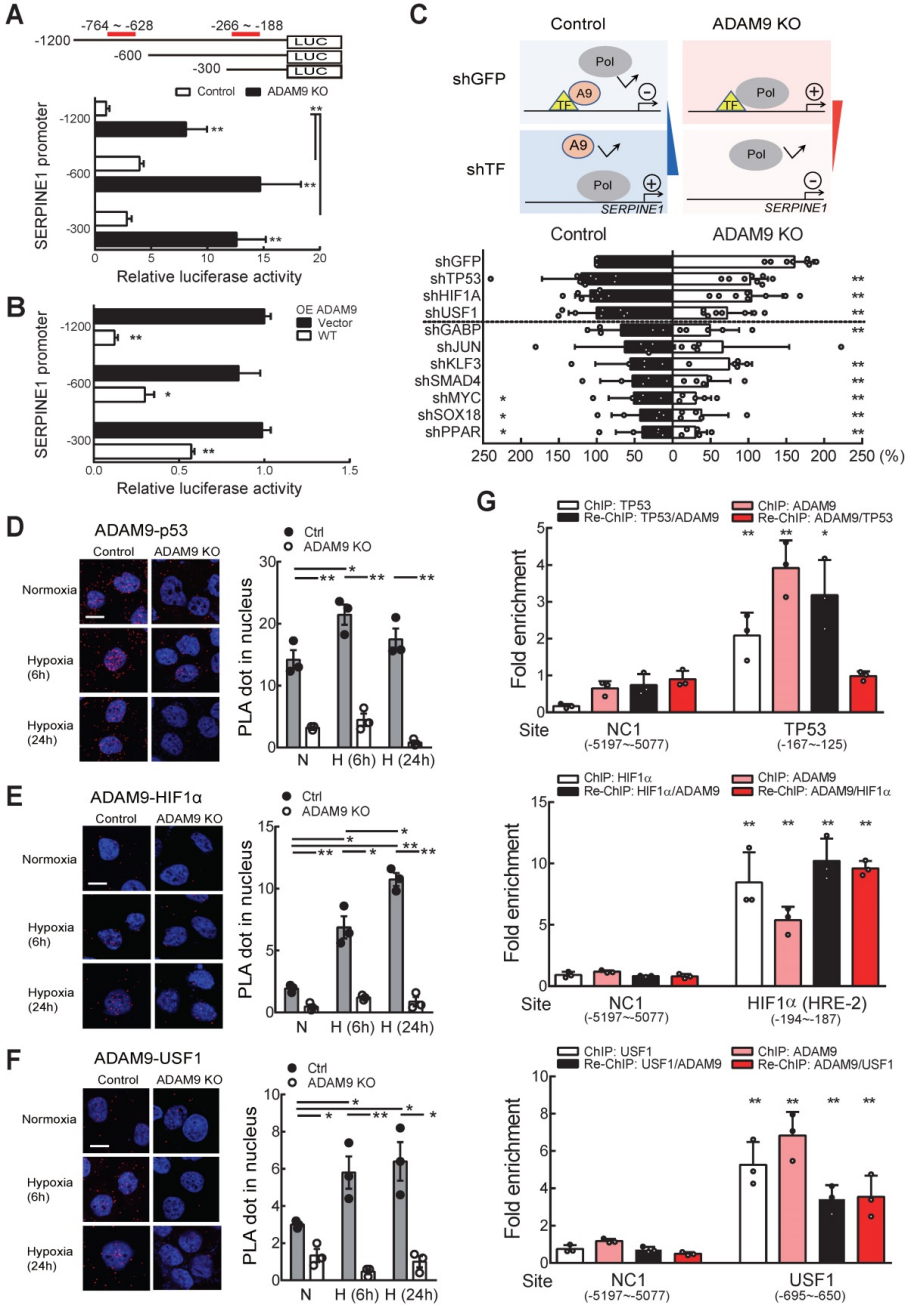
** Nuclear ADAM9 proteins interact with transcription factors HIF1α and USF1 in ESCC cells. (A)** *SERPINE1* promoter activity in control and *ADAM9* KO KYSE170 cells. A schematic diagram of *SERPINE1* promoter constructs from sites -1200, -600, or -300 driving luciferase expression (top). The two previously reported repressor sites are marked in red. Relative luciferase activity was shown by Firefly/Renilla luciferase ratio in the dual-luciferase assay. **(B)** The transcriptional activity of the indicated promoters of *SERPINE1* in *ADAM9* KO KYSE170 cells with overexpression of ADAM9 WT. **(C)** Identification of potential transcription factors involved in ADAM9-mediated transcriptional suppression of *SERPINE1* in KYSE170 cells. Schematics of transcriptional responses of *SERPINE1* regulated by the interaction of ADAM9 and transcription factor (TF) (top). A9, ADAM9; Pol, RNA polymerase. The shRNA of TF was transiently transfected in control and ADAM9 KO cells and then cells were subjected to assay for the luciferase activity (bottom). Error bar, ±s.d. **(D-F)** PLA detection of ADAM9 and transcription factors in KYSE170 cells. Duolink PLA analysis of ADAM9/p53 complexes (**D**); ADAM9/HIF1α complexes (**E**); ADAM9/USF1 complexes (**F**). Representative images of PLA (red) and nuclei (DAPI, blue). Quantification of PLA was analyzed from 3 fields. Scale bars, 10 µm. Statistical analysis was performed with a t-test. **(G)** KYSE170 cells were ChIP and re-ChIP using indicated transcription factor and ADAM9 antibodies sequentially or in reverse order. Chromatin samples were analyzed by qPCR at the transcription factor binding sites. A t-test was compared with the NC1 group. *, p < 0.05; **, p < 0.01.

Next, we investigated whether ADAM9 influenced *SERPINE1* gene transcription by measuring *SERPINE1* promoter activity. Two repressor elements (-764 to -628 and -266 to -188) have been previously identified in the *SERPINE1* promoter, but the identity of potential repressors remains unknown 28. We linked the 5'-flanking regions of the *SERPINE1* promoter at positions -1200, -600, and -300, respectively, with the luciferase gene to measure the promoter activity in control and *ADAM9* KO cells (Figure 6A). *SERPINE1* promoter activity was greater in *ADAM9* KO cells compared to control cells for all three promoter constructs, but the two shorter constructs (-600 and -300) showed greater promoter activity than the longest construct (-1200) (Figure 6A). This is likely due to the presence of an unknown repressor sequence located within the region of -1200 to -600 bp. Similar observations of *SERPINE1* promoter activity were found in *ADAM9* knockdown ESCC cells (Figure S6A). Moreover, in *ADAM9* KO KYSE170 cells, overexpression of ADAM9 WT strongly reduced the promoter activity of *SERPINE1* compared with vector controls (Figure 6B). Although *SERPINE1* promoter contains a hypoxia-responsive element 29, its activity remains low under stressed condition of hypoxia with starvation in ADAM9-expressing shGFP cells (Figure S6B). In contrast, ADAM9 deficiency resulted in activation of *SERPINE* promoter under stressed conditions (Figure S6B). These results suggest that ADAM9 plays an important role in the negative regulation of *SERPINE1* gene expression.

Because ADAM9 has no DNA binding domain, we speculate that it may be recruited by known transcription factors and then suppress their transactivation. To screen for the potential ADAM9-interacting transcription factors, we evaluated the promoter activity in control and ADAM9 KO cells by transiently knocking down known transcription factors involved in *SERPINE1* gene regulation. We reason that the transcriptional inhibitory effect of ADAM9 for *SERPINE1* gene would be reversed upon additional knockdown of ADAM9-interacting transcription factors in control cells based on the assumption that ADAM9 may not be able to be recruited by these transcription factors to the promoter region. In contrast, in the absence of ADAM9 (in ADAM9 KO cells), these transcription factors may increase the transactivation of *SERPINE1* promoter and this would be reversed upon further knockdown of transcription factors (Figure 6C, top). Consistent with our prediction, we found that knockdown of transcription factor TP53, HIF1α, and upstream transcription factor 1 (USF1) in control cells showed elevated transactivation of* SERPINE1* promoter whereas the promoter activity in ADAM9 KO cells was reduced (Figure 6C, bottom). The other transcription factors we screened may not be related to ADAM9 transcriptional regulation in *SERPINE1* promoter based on our observations that their knockdown non-selectively decreased the promoter activity in control and ADAM9 KO cells (Figure 6C, bottom). To validate whether ADAM9 can interact with these transcription factors in a physiologically relevant manner, we conducted a sensitive proximity ligation assay (PLA), which can detect the close proximity of two molecules in the cells using two primary antibodies. We found that PLA signals of ADAM9 and TP53 were elevated in the whole cell and the nucleus of control cells during hypoxia (Figure 6D and Figure S6C). Similar phenomenon was detected in PLA signals of ADAM9 and HIF1α (Figure 6E and Figure S6C), as well as those of ADAM9 and USF1 (Figure 6F and Figure S6C). Re-ChIP analysis of sequential immunoprecipitation of two chromatin-binding proteins can be used to study co-occupancy of two factors on a specific DNA sequence 30. In single ChIP analysis, either individual known transcription factor or ADAM9 bound well to the *SERPINE1* promoter (Figure 6G). In re-ChIP analysis, we showed that the three transcription factors and ADAM9 were co-enriched on their binding sites at *SERPINE1* promoter (Figure 6G). Similar levels of co-enrichment in HIF1α (first IP)/ADAM9 (second IP) and ADAM9 (first IP)/HIF1α (second IP) indicated that HIF1α and ADAM9 co-occupied the *SERPINE1* promoter. Notably, a higher level of enrichment is seen with TP53/ADAM9 than ADAM9/TP53, suggesting that these two proteins show partial co-occupancy: TP53 can co-bind with ADAM9, but ADAM9 may not always co-bind with TP53 to the *SERPINE1* promoter. Co-occupancy of USF1 and ADAM9 was also detected with reciprocal re-ChIP at known USF1 binding site (-695 ~ -650) of *SERPINE1* promoter. Taken together, these results suggest that ADAM9 proteins interact with selective transcription factors to dampen transcription of *SERPINE1* gene in ESCC.

## Discussion

**Figure 7:**
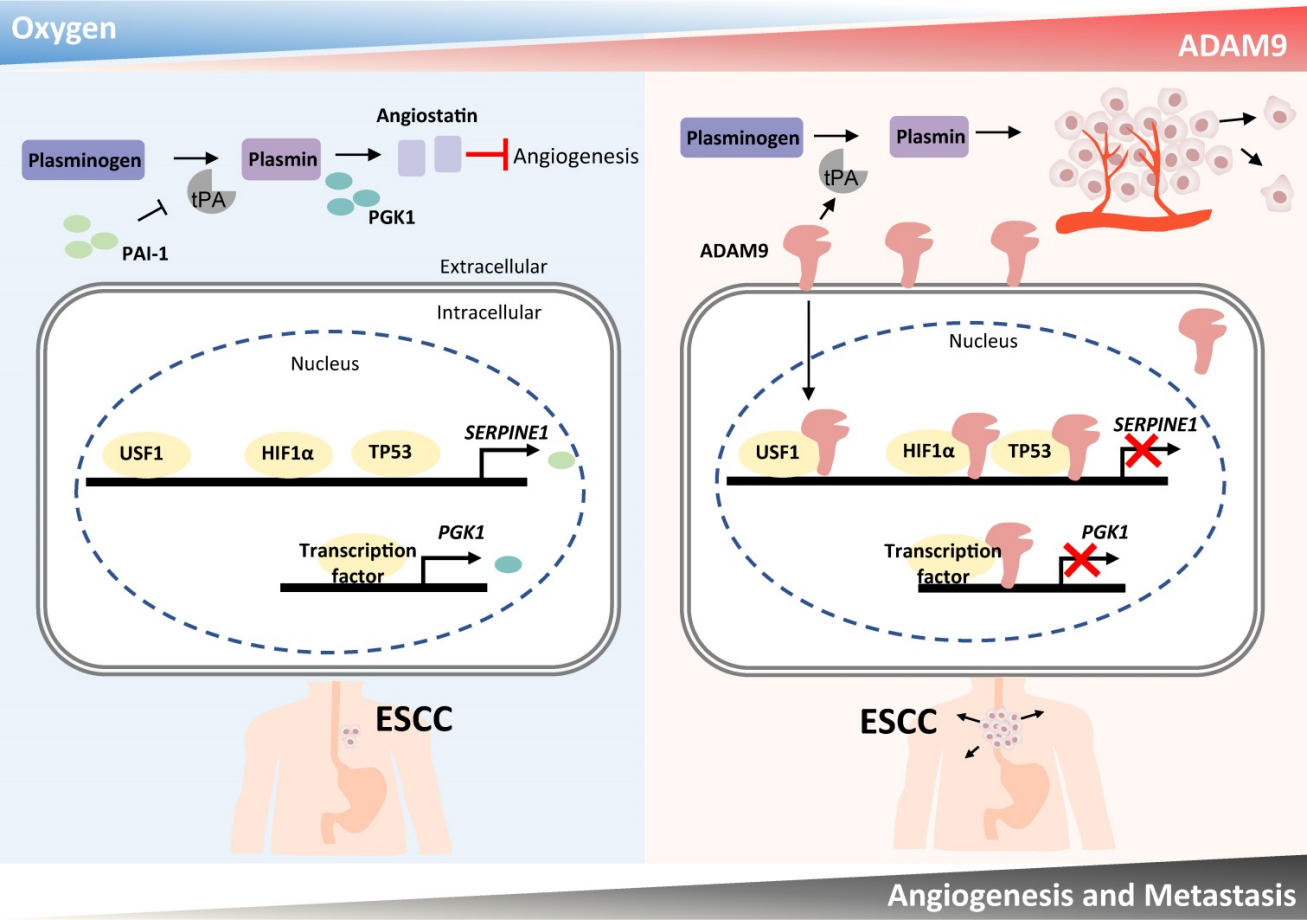
**Schematic diagram showing a dual role of ADAM9 in angiogenesis regulation through its protease activity and transcriptional regulation in nucleus.** The protease function of ADAM9 increases *PLAT* expression for extracellular matrix degradation and proangiogenesis. In hypoxia, nuclear translocation of ADAM9 is increased and nuclear ADAM9 occupies the promoters of *SERPINE1* and *PGK1*, leading to transcriptional repression of *SERPINE1* and *PGK1* for reducing angiostatin formation and thus promoting angiogenesis.

Here we have demonstrated that a high level of ADAM9 is correlated with poor outcomes in ESCC patients. Silencing of ADAM9 reduced tumorigenic characteristics of ESCC *in vitro* and *in vivo*, such as invasive and metastatic ability, and distant metastasis. We propose that ADAM9 coordinates plasminogen activator-based network for ESCC progression through two major functions of ADAM9: protease activity for increasing tPA expression/activity and nuclear translocation of ADAM9 for suppressing PAI-1 and PGK1 expression (PAI-1 for reducing plasmin and PGK1 for increasing angiostatin), two inhibitory factors involved in plasminogen activator-mediated angiogenesis. Thus, we revealed a novel role of ADAM9 in promoting angiogenesis through a previously unappreciated mechanism of transcriptional repression of genes participating in the negative regulation of angiogenesis (Figure 7).

The sheddase function of ADAM9 has been considered critical for promoting cell proliferation and migration in ESCC through yielding soluble EGFR ligands, thereby activating the EGFR-AKT pathway 18. Here we demonstrated that the sheddase activity of ADAM9 is also involved in increasing tPA protein expression and activity. Importantly, protease-defective ADAM9 can reduce its nuclear translocation. We found that nuclear ADAM9 can occupy the *SERPINE1* promoter over nearly 1 kb and suppress *SERPINE1* transcription. We provide evidence suggesting that ADAM9 is likely to interact with several transcription factors previously known for regulation of *SERPINE1* expression, such as p53, HIF1α, and USF1. These transcription factors play important roles in tumorigenesis and interplay with each other to regulate target gene expression. They serve as positive regulators by binding to *SERPINE1* promoter to activate PAI-1 gene expression under various contexts. Beyond the role as a TF, the tumor suppressor p53 binds to PAI-1 mRNA 3'-UTR and stabilizes PAI-1 mRNA, leading to enhanced PAI-1 expression 31. The stress sensor USF1 not only binds to and transactivates TP53 promoter 32, but also stabilizes p53 proteins to coordinate p53 function in cell fate decision 33. Loss of USF1 drives p53 degradation and accelerates gastric carcinogenesis 34. p53 influences HIF-1α by suppressing its hypoxic induction and target activation, while p53 loss of function enhances HIF-1α in tumors 35. Conversely, severe and prolonged hypoxic stress upregulates p53 for cell fate decision 36. Upregulation of HIF-1α activates crucial cancer hallmarks such as angiogenesis leading to poor cancer patient prognosis. Altogether, p53, HIF1α, and USF1 portray a complicated regulation network on multiple target genes, including PAI-1, suggesting that PAI-1 level needs to be critically regulated in maintaining cell function. Our study uncovers ADAM9 as a new player of PAI-1 regulation network, presumably through protein-protein interaction between ADAM9 and the above mentioned transcription factors. However, whether and how the interaction of ADAM9 with these transcription factors regulate other genes or pathways during cancer progression need to be clarified in future studies.

Previous studies showed positive nuclear staining of ADAM9 and its association with lethal phenotypic progression of prostate cancer 37, but the role and regulation of nuclear ADAM9 has not been elucidated. Our finding reveals that ADAM9 can be induced upon hypoxia to translocate to the nucleus, where nuclear ADAM9 acts as a transcriptional repressor to regulate expression of key angiogenesis genes in ESCC cells. Another member of the ADAM family, ADAM10, has been reported to be transported into the nucleus and contributes to the pathogenesis and progression of human prostate cancer 38. Although the overall sequence similarity between ADAM9 and ADAM10 is about 25%, the putative nuclear localization sequence (NLS, aa 199 to 206) in ADAM9 is highly similar (87.5%) to the NLS region (aa 207 to 214) in ADAM10. Whether the putative NLS of ADAM9 mediates protein nuclear translocation in a similar manner as that of ADAM10 is worth further investigation.

Angiogenesis contributes to the aggressive characteristics of ESCC 39. The plasminogen activator-based pathway promotes tumor-associated angiogenesis via protease activity 9. Although PAI-1 has been considered as an inhibitor of cancer development, the proangiogenic and promigratory effects of PAI-1 promote tumor growth at physiological concentration by stimulating plasmin-mediated proteolysis 40. Several studies have revealed that elevated levels of PAI-1 in tumor tissue are predictors of poor patient outcomes in the subset of breast cancer patients with lymph node-negative disease 41. However, genetic ablation of PAI-1 had no effects on tumor development or metastasis 42. Given that a typical rapid decline in PAI-1 levels was detected prior to the onset of DNA synthesis 43 and PAI-1 reduction leading to escape from senescence-associated proliferative arrest in primary fibroblasts 44, PAI-1 may have a negative influence on cell growth. Indeed, at supraphysiological levels, PAI-1-mediated inhibition of tumor vascularization was observed in either host cells or tumor cells 45, suggesting that high levels of PAI-1 reduce angiogenesis. Our current work provides new insights into how ADAM9 regulates tumor-associated angiogenesis in a context-dependent manner. Under stressed conditions, nuclear ADAM9 may function as a transcriptional repressor to maintain PAI-1 at a low level. Consistent with this concept, our data show that stressed conditions enhance a strong induction of PAI-1 expression only in ADAM9-deficient cells, but not in ADAM9-abundant cells. Taken together, we reveal a novel role of ADAM9 as a stress-induced transcriptional regulator to undermine PAI-1 expression to promote tumor vascularization.

## Supplementary Material

Supplementary methods, figures and tables.

## Abbreviations

ADAM9: a disintegrin and metalloprotease 9
ChIP: chromatin immunoprecipitation
EGFR: epidermal growth factor receptor
ESCC: esophageal squamous cell carcinoma
HIF1α: hypoxia inducible factor 1 subunit alpha
IHC: immunohistochemistry
KO: knockout
PAI-1: plasminogen activator inhibitor type I
PGK1: phosphoglycerate kinase 1
PLA: proximity ligation assay
SERPINE1: serpin family E member 1
TIC: tumor-initiating cell
tPA: tissue plasminogen activator
TCGA: The Cancer Genome Atlas
WT: wild type
