# Supplementary material for: ADAM9 functions as a transcriptional regulator to drive angiogenesis in esophageal squamous cell carcinoma
Source: Int J Biol Sci. 2021 Sep 7;17(14):3898–910. doi: 10.7150/ijbs.65488 (PMC8495400; doi:10.7150/ijbs.65488)
Supplement: Supplementary file 1 — Supplementary methods, figures and tables. [file ijbsv17p3898s1.pdf]

Supplementary Information

ADAM9 functions as a transcriptional regulator to drive angiogenesis in esophageal squamous cell carcinoma

Yu-Sen Lin, Ting-Ting Kuo, Chia-Chien Lo, Wei-Chung Cheng, Wei-Chao Chang, Guan-Chin Tseng, Shih-Ting Bai, Yu-Kai Huang, Chih-Ying Hsieh, Han-Shui Hsu, Yi-Fan Jiang, Chen-Yuan Lin, Liang-Chuan Lai, Xing-Guo Li, and Yuh-Pyng Sher*

*Correspondence should be addressed to Yuh-Pyng Sher, E-mail: ypsher@mail.cmu.edu.tw

Includes:

1. Supplementary Methods
2. Supplementary References
3. Supplementary table
4. Supplementary figures (6 Figures)

Supplementary Methods

GEO and TCGA dataset analysis

Gene Expression Omnibus (GEO) dataset (GSE20347) was used to analyze the differentially expressed genes in normal and tumor specimens by GEO2R platform (https://www.ncbi.nlm.nih.gov/geo/geo2r/?acc=GSE20347). Detailed information on the processing of the TCGA dataset was described in our previous studies [1]. Samples of the TCGA ESCC dataset were selected for analysis in this study.

Cell culture and reagents

The Food Industry Research and Development Institute (Hsinchu City, Taiwan; http://www.firdi.org.tw/index.htm) provided the esophageal cancer cell lines CE146T/VGH (BCRC 60167), CE81T/VGH (BCRC 60166), and CE48T/VGH (BCRC 60165), originally generated at the Taipei Veterans General Hospital. The cell line KYSE170 was kindly provided by Professor Yi-Ching Wang (National Cheng Kung University). All cell lines were free from *Mycoplasma* contamination.

The following antibodies were used in western blot: anti-ADAM9 (AF939, R&D System), anti-CDCP1 (ab1377, Abcam, Cambridge, MA), anti-t-PA (103453, Gentex), anti-pEGFR-Y1068 (3777, Cell signaling), EGFR (SC373746, Santa Cruz Biotechnology), anti-pAKT-Ser473(9271, Cell signaling), AKT (2920, Cell signaling), anti-EF1α (05-235, Millipore, Billerica, MA), anti-PAI-1 (sc-5297, Santa Cruz Biotechnology), anti-HIF1α (MAB1536, R&D System), anti-HA (H3663, Sigma). The anti-ADAM9 antibody from Cell Signaling, (#2099; Danvers, MA) was used to detect two forms of ADAM9 (about 80 and 120 kD).

Immunohistochemical staining

IHC analysis was performed to study the expression of ADAM9 in human ESCC samples as previously described [2]. Briefly, goat anti-mouse ADAM9 antibody (AF949, R&D systems, Wiesbaden, Germany) was used to perform IHC staining using horseradish peroxidase-conjugated avidin-biotin complex (ABC) in a Vectastain Elite ABC Kit (Vector Laboratories, Burlingame, CA, USA). Tissue sections were counterstained with hematoxylin and mounted. The staining was evaluated by pathologists who were unaware of the clinicopathological parameters and clinical outcomes of the patients.

RNA-sequencing and analysis

The RNA-sequencing of control and ADAM9 KO KYSE170 samples were performed by Welgene Biotech Co. Ltd. (Taipei, Taiwan). Briefly, total RNA was extracted by Trizol Reagent (Invitrogen, USA) and the RNA quality was measured using a Bioanalyzer 2100 with a RNA 6000 LabChip kit (Agilent Technologies, USA) with an RNA Integrity Number ≥ 7. The library was constructed using Agilent's SureSelect Strand-Specific RNA Library Preparation Kit for 2X150bp (Paired-End) and AMPure XP beads (Beckman Coulter, USA) size selection. The sequence was determined using Illumina's sequencing-by-synthesis (SBS) technology (Illumina, USA) NovaSeq. The sequencing results (FASTQ reads) were generated using Welgene Biotech's pipeline based on Illumina's base calling program bcl2fastq v2.20. We further performed functional enrichment analysis of the differential expression genes as previously described [3].

Quantitative reverse transcription PCR

Quantitation of ADAM9, PLAT, and SERPINE1 was performed as previously described [4]. The following primer pairs were used for mRNA detection: *PGK1*: 5’-GCCAGTTGCTGTAGAACTCAAA-3’ (forward), 5’-GGGCCTACACAGTCCTTCAA-3’ (reverse). The relative expression level of mRNA was normalized against *GAPDH* mRNA.

Gene knockdown and knockout in ESCC cells

Lentiviral shRNA targeting ADAM9, SERPINE1, and PLAT was used as previous described [4] and reagent were obtained from the National RNAi Core Facility, Institute of Molecular Biology, Genomic Research Center, Academia Sinica. The human library is referred to as TRC-Hs 1.0. ESCC cells were infected with lentiviral suspensions and selected with 2 μg/ml puromycin to knock down ADAM9 expression. The efficiency of the gene knockdown was validated by Western blot analysis. A CRISPR RNA-guided Cas9 nuclease gene targeting system was used to knock out the ADAM9 gene in ESCC cells with a ToolGen kit (ToolGen Inc.) as previously described [5].

Plasmids, transfection, and generation of stable cell lines

The plasmids of HA-ADAM9 WT and catalytic mutant E348A were constructed as previously described [5]. The different fragments of *SERPINE1* promoter were constructed in the luciferase reporter plasmid pGL3-basic with MluI and XhoI sites, and then they were co-transfected with *Renilla* luciferase reporter plasmids into the indicated cells for detecting promoter activity using the Dual-Luciferase Reporter Assay System (Promega, Madison, WI, USA).

tPA protein and activity assay

Cells were seeded on 6cm dishes at 80-90% confluency. After 24h incubation, the conditioned media were harvested by centrifuging at 3000g. The tPA protein concentration and activity in conditioned media were measured by a Human Total tPA ELISA Kit (ET2001-1, Assaypro LLC, St. Charles, MO, USA) and a Human tPA Chromogenic Activity Kit (CT1001, Assaypro LLC, St. Charles, MO, USA), respectively. All experiments were performed according to the manufacturer's instructions.

Time-lapse migration assay

This assay was conducted as previously described [4]. Briefly, cells were cultured on collagen-coated dishes (10 μg/mL, 3 mL) in serum-free media. The migration of cells was captured with CCD video cameras (AxioCam MRm, Zeiss) at 20 min intervals for a total 16 hours by inverted microscopes (Axio Observer Z1, Zeiss). Accumulated migration distance was determined by using the Track Point function of Image J.

Endothelial tube formation

The detailed procedures have been previously described [5]. Briefly, Matrigel was added to each well of a μ-slide (ibidi GmbH, Munich, Germany) and allowed to polymerize for 30 min at 37 °C. Cell suspensions (50 μL) of HUVECs treated with conditioned media from the indicated cells were plated in the Matrigel-coated wells at 7500 cells/well in serum-free M199 medium for 4 h at 37 °C in a 5% CO_2 humidified atmosphere incubator. After 4-h incubation, capillary-like structures formed, and the images were captured using a Cytation™ 5 plate reader (BioTek, Winooski, VT, USA). The images were analyzed by measuring the total length of the tubules per well with ImageJ software (National Institutes of Health, Bethesda, MD, USA).

ChIP-sequencing and ChIP-qPCR

Cultures of 2×10^7 ESCC cells were fixed in 1% formaldehyde for 10 min at room temperature. After quenching the reaction by 0.25 M glycine, the cells were scraped in 1 mL cell lysis buffer (50 mM HEPES at pH 7.5, 150 mM NaCl, 1 mM EDTA, 1% Triton X-100, 0.1% sodium deoxycholate, 0.1% SDS, and protease inhibitors) and lysed by dounce homogenization. Nuclear pellets were collected and lysed in 0.5 mL nuclear lysis buffer (50 mM HEPES at pH 7.5, 150 mM NaCl, 1 mM EDTA, 1% Triton X-100, 0.1 % sodium

deoxycholate, 1% SDS, and protease inhibitors). Nuclear lysates were sonicated with a Bioruptor (Diagenode) to shear the chromatin DNA to ~0.2-0.5 kb in length. The sheared chromatin was diluted 10 times with ChIP dilution buffer (0.01% SDS, 1.1% Triton X-100, 1.2 mM EDTA, 16.7 mM Tris-HCl, pH8.0, 167mM NaCl, protease inhibitor) and incubated with anti-ADAM9 antibody (AF939, R&D systems) and protein A/G magnetic beads (Invitrogen) overnight at 4°C on a rotator. Wash each IP sample once with wash buffer I (20 mM Tris–Cl pH 8.0, 150 mM NaCl, 2 mM EDTA, 1% Triton X-100, 0.1% SDS), II (20 mM Tris–Cl pH 8.0, 500 mM NaCl, 2 mM EDTA, 1% Triton X-100, 0.1% SDS), III (250 mM LiCl, 10 mM Tris–Cl pH 8.0, 1% sodium deoxycholate, 1 mM EDTA, 1% Igepal CA-630) and 1x TE (10 mM Tris–Cl pH 8.0, 1 mM EDTA) buffer, sequentially. After washing, the protein A/G bead-antibody/chromatin complex was treated with final 200 μg/mL proteinase K (Biokit) and final 0.3 M NaCl in elution buffer (50 mM $NaHCO_3$, 1% SDS) at 65°C overnight. All samples were eluted by incubation at 95°C for 10 min, followed by extraction with phenol:chloroform:isoamyl alcohol and ethanol precipitation at -80°C. The ChIP DNA was resuspended in water for further sequencing.

The re-ChIP assays were conducted based on ChIP assays by previous protocols [6]. Briefly, the chromatin samples were harvested and incubated with indicated antibody and dynabeads protein A/G (Invitrogen) at 4°C overnight on a rotator for the 1st IP. Wash each 1st IP sample once with wash buffer I, II, III and 1x TE buffer, sequentially. Elute the 1st IP protein-DNA complexes by adding re-ChIP elution buffer (1X TE, 2%SDS, 15 mM DTT, protease inhibitor). For the 2nd IP, the eluted samples were diluted 20 times with ChIP dilution buffer and were incubated with the indicated antibody and dynabeads protein A/G at 4°C overnight on a rotator. All the 2nd IP samples were washed with wash buffer and eluted by elution buffer (50mM NaHCO3, 1% SDS). To reverse protein-DNA cross-link, all samples (input and IP) were added with 5M NaCl to a final concentration of 0.2M and 200 μg/mL protease K and incubated at 65°C for 4-5hr or overnight, followed by extraction with

phenol:chloroform:isoamyl alcohol and ethanol precipitation at -80 °C. The ChIP DNA was resuspended in water for further qPCR assay. The anti-ADMA9 (AF939, R&D), anti-USF1 (sc390027, Santa Cruz), anti-TP53 (2524, Cell Signaling Technology) anti-HIF1α (610959, BD), mouse IgG isotype control (GTX35009, GeneTex) and goat IgG isotype control (I5256, Sigma) were used for re-ChIP assays.

The following primer pairs were used for *SERPINE1* promoter detection: pair 1: 5'-TTGAATCATCCCGAAACCAT-3' (forward), 5'- GGGAGGAAGAGTGACCGATA-3' (reverse); pair 2: 5'- AACTTACACGTTGGTCTCCTGT -3' (forward), 5'-GTCAACAACCTTGTCTGGCT -3' (reverse); pair 3: 5'- GGGAGTCAGCCGTGTATCAT -3' (forward), 5'- GCCAGCCACGTGATTGTCTA -3' (reverse); pair 4: 5'-TAACTCCACAGTGACCTGGTTC -3' (forward), 5'- GTTGACTGTCTGCCATGCCG -3' (reverse); pair 5: 5'- TTCTCAGGCCAAGGCTATTG -3' (forward), 5'-CTCTGGGAGTCCGTCTGAAC -3' (reverse); pair 6: 5'- GTGGGGCTGGAACATGAGTT-3' (forward), 5'- TGTGGGTCTTCTTGACAGCG -3' (reverse); pair 7 (USF1 binding site) : 5'- GTTCAGCCACCACCACCCCA -3' (forward), 5'- TGGTCTTTCCCTCATCCCTGC-3' (reverse); pair 8 (TP53 binding site) : 5'- GTCAAGGGAGGTTCTCAGGC -3' (forward), 5'-ACCTCCCTCTCTGGGACTTG-3' (reverse); pair 9 (HRE-2, HIF1α binding site) : 5'-ACAGACACAGGCAGAGGGCA -3' (forward), 5'- TGTGTGTGTGTGTGTGCTGCT -3' (forward); and the negative control (NC) pair: 5'- CCAGCACTGGTTCCCTCCTA -3' (forward), 5'- AGGCAGGGTCTTGCTAACAG -3' (reverse). Fold enrichment was calculated by determining the apparent immunoprecipitation efficiency (ratios of the amount of immunoprecipitated DNA over that of the IgG control) and normalized to the level observed at the NC control region, which was defined as 1. DNA segments enriched by the re-ChIP are then measured by quantitative PCR (qChIP). The two parallel re-ChIPs are carried out in reverse order for the first and second protein and then compared with each

single qChIP to determine full, partial, or no co-occupancy of the two proteins on the same region.

Reference

1. Chung IF, Chang SJ, Chen CY, et al. YM500v3: a database for small RNA sequencing in human cancer research. Nucleic Acids Res. 2017; 45: D925-D31.
2. Sher Y-P, Tzeng T-F, Kan S-F, et al. Cancer targeted gene therapy of BikDD inhibits orthotopic lung cancer growth and improves long-term survival. Oncogene. 2009; 28: 3286-95.
3. Cheng WC, Chang CY, Lo CC, et al. Identification of theranostic factors for patients developing metastasis after surgery for early-stage lung adenocarcinoma. Theranostics. 2021; 11: 3661-75.
4. Lin CY, Chen HJ, Huang CC, et al. ADAM9 promotes lung cancer metastases to brain by a plasminogen activator-based pathway. Cancer Res. 2014; 74: 5229-43.
5. Lin CY, Cho CF, Bai ST, et al. ADAM9 promotes lung cancer progression through vascular remodeling by VEGFA, ANGPT2, and PLAT. Sci Rep. 2017; 7: 15108.
6. Peng GH, Chen S. Double chromatin immunoprecipitation: analysis of target co-occupancy of retinal transcription factors. Methods Mol Biol. 2013; 935: 311-28.

Supplementary Table

Table S1. Clinicopathological features of 213 ESCC patients

Clinicopathological features		Total	ADAM9 expression		P value
			Negative (%)	Positive (%)	
**All cases**		213	119 (55.9%)	94 (44.1%)	
**Age**	＜ 60	90	51 (56.7%)	39 (43.3%)	0.89
	≧ 60	123	68 (55.3%)	55 (44.7%)	
**Sex**	Male	202	112 (55.4%)	90 (44.6%)	0.75
	Female	11	7 (63.6%)	4 (36.4%)	
**T**	1,2	61	38 (62.3%)	23 (37.7%)	0.29
	3,4	152	81 (53.3%)	71 (46.7%)	
**N**	0	86	41 (47.7%)	45 (52.3%)	0.05
	1	127	78 (61.4%)	49 (38.6%)	
**M**	0	183	107 (58.5%)	76 (41.5%)	0.07
	1	30	12 (40%)	18 (60%)	

Table S2. Top 10 specific peaks of ADAM9 ChIP-sequence

Symbol	Chromosome	ChIP start	ChIP end	Width	Score	Distance to TSS	Annotation	Gene name
SERPINE1	chr7	100769634	100770663	1030	87.87768	0	Promoter (<=1kb)	serpin peptidase inhibitor, clade E (plasminogen activator inhibitor type 1), member 1
BASP1	chr5	17275328	17276009	682	155.5665	57658	Exon 2	brain abundant, membrane attached signal protein 1
GH1	chr17	61994058	61994668	611	103.18775	1530	3' UTR	growth hormone 1
AP3M1	chr10	75878951	75879264	314	28.74207	31255	3' UTR	adaptor-related protein complex 3, mu 1 subunit
HLA-C	chr6	31236638	31236938	301	26.01127	2187	3' UTR	major histocompatibility complex, class I, C
FAM53A	chr4	1601252	1601459	208	25.94286	84259	Distal Intergenic	family with sequence similarity 53, member A
MIR3620	chr1	228287795	228288061	267	19.80584	2831	Downstream (<1kb)	microRNA 3620
CACNG2	chr22	37099096	37099519	424	13.29284	-406	Promoter (<=1kb)	calcium channel, voltage-dependent, gamma subunit 2
SCTR	chr2	120281708	120281942	235	9.63757	86	Promoter (<=1kb)	secretin receptor
PGK1	chrX	77359368	77359748	381	7.32426	0	Promoter (<=1kb)	phosphoglycerate kinase 1

Supplementary Figures

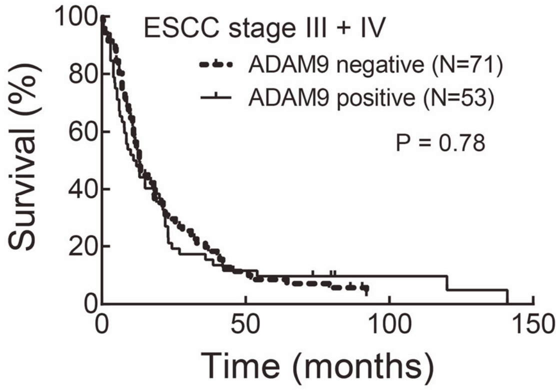

Figure S1, related to Figure 1. Kaplan-Meier survival curve of ESCC patients with advanced stage (stage III and IV) grouped by ADAM9 IHC staining status (positive or negative).

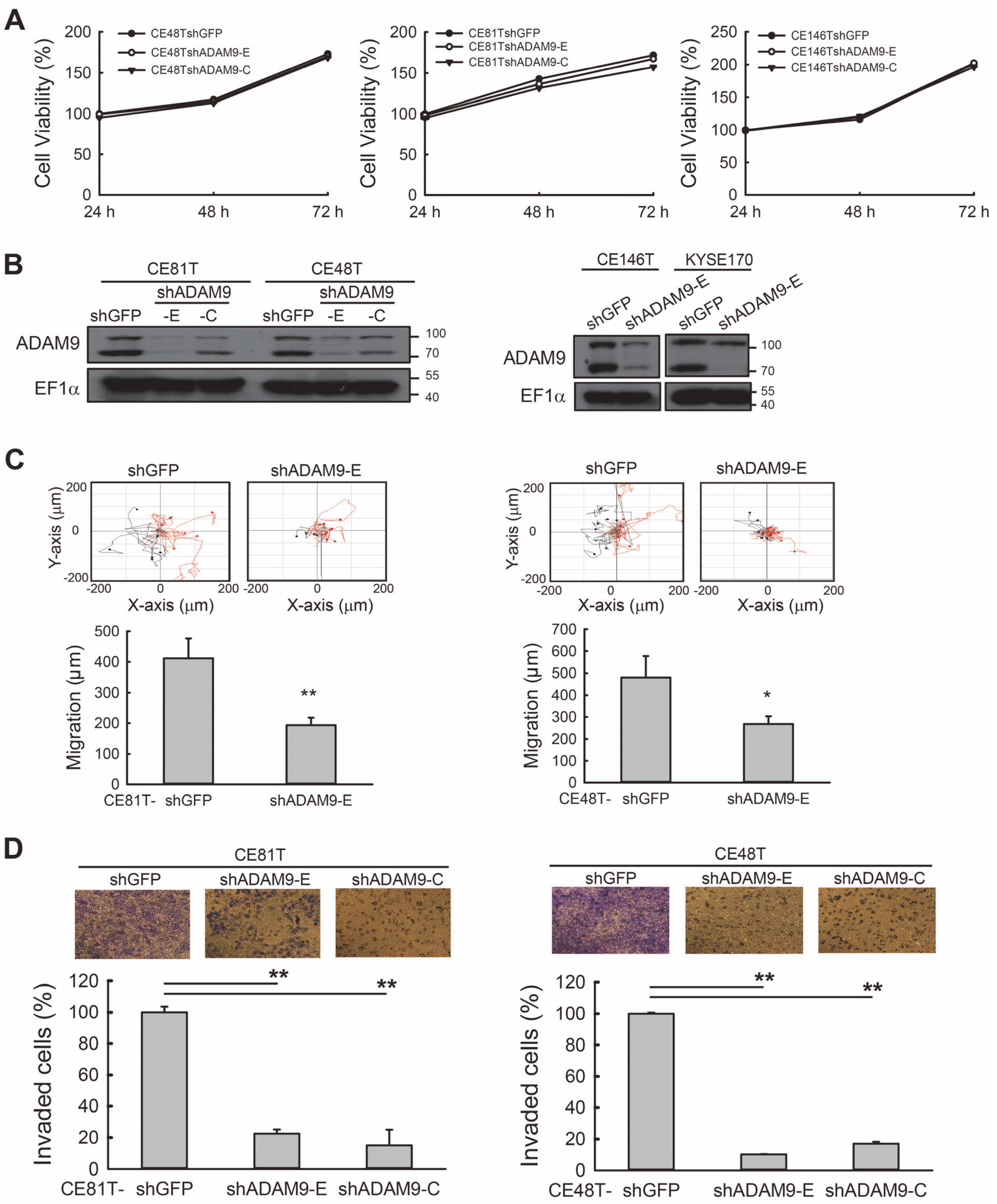

Figure S2, related to Figure 2. **(A)** The cell proliferation rate of control (shGFP) and ADAM9 knockdown (shADAM9) cells by MTT assay. Error bar, SD. **(B)** Western blot analysis of ADAM9 in control (shGFP) and *ADAM9* knockdown (shADAM9-E, shADAM9-C) ESCC cells. **(C)** Videomicroscopy of individual cells is quantitated to determine the distance and direction of cellular migration of ESCC cells with either control or ADAM9 knockdown CE81T and CE48T cells. **(D)** Crystal violet dye staining of control and ADAM9 knockdown

CE81T and CE48T cell lines that migrated in the transwell assays with Matrigel after 2 days. Data are mean ± SD. *, $P < 0.05$. **, $P < 0.01$.

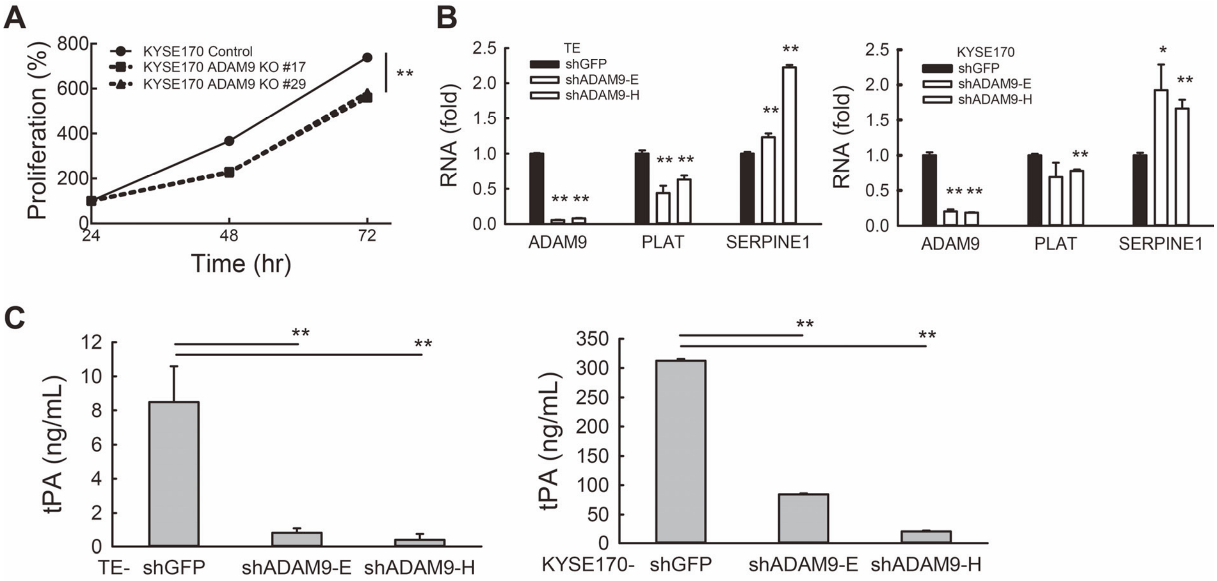

Figure S3, related to Figure 3. **(A)** The cell proliferation rate of control and two clones of ADAM9 knockout (KO) cells by MTT assay. Error bar, SD. **(B)** RT-qPCR of *ADAM9*, *PLAT*, and *SERPINE1* in control and *ADAM9* knockdown TE cells and KYSE170 cells. **(C)** tPA was detected in the culture media of TE cells and KYSE170 cells.

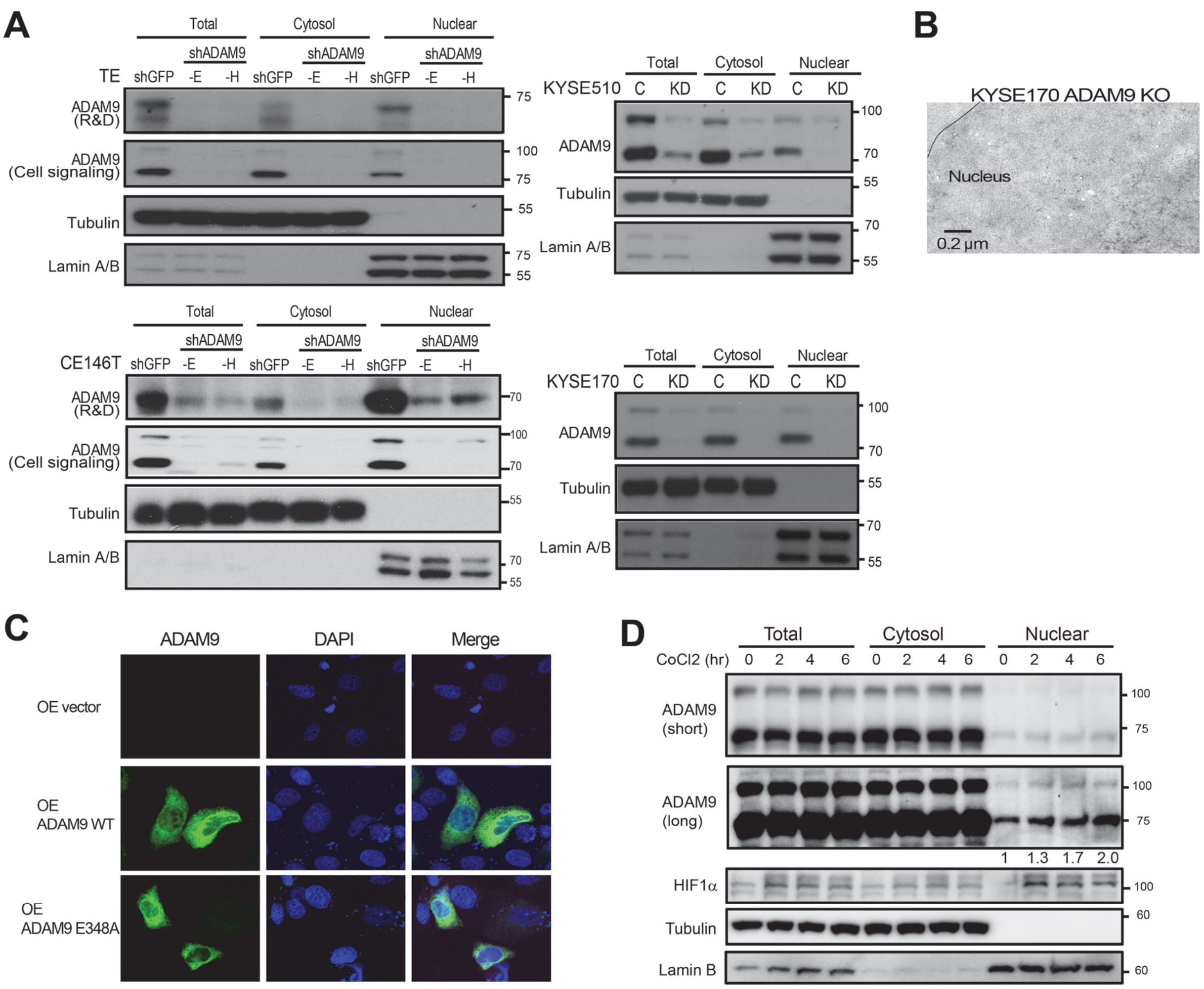

Figure S4, related to Figure 4. **(A)** Western blot analysis of cellular localization of ADAM9 in control and *ADAM9* knockdown ESCC cells. Markers for cytoplasm and nucleus are α-tubulin and lamin A/B, respectively. ADAM9 antibodies from R&D and Cell Signaling were used for N-terminal and C-terminal domain detection, respectively. **(B)** No signal of ADAM9 was detected in ADAM9 KO KYSE170 cells using TEM. **(C)** Fluorescence immunohistochemistry of ADAM9 in *ADAM9* KO KYSE170 cells transiently transfected with plasmids of ADAM9 with confocal microscopy. **(D)** Cellular fractionation followed by western blot analysis of KYSE170 cells treated with 300 μM $CoCl_2$ at different time points. Numbers beneath each panel indicate the amount of protein relative to the 0 h time point.

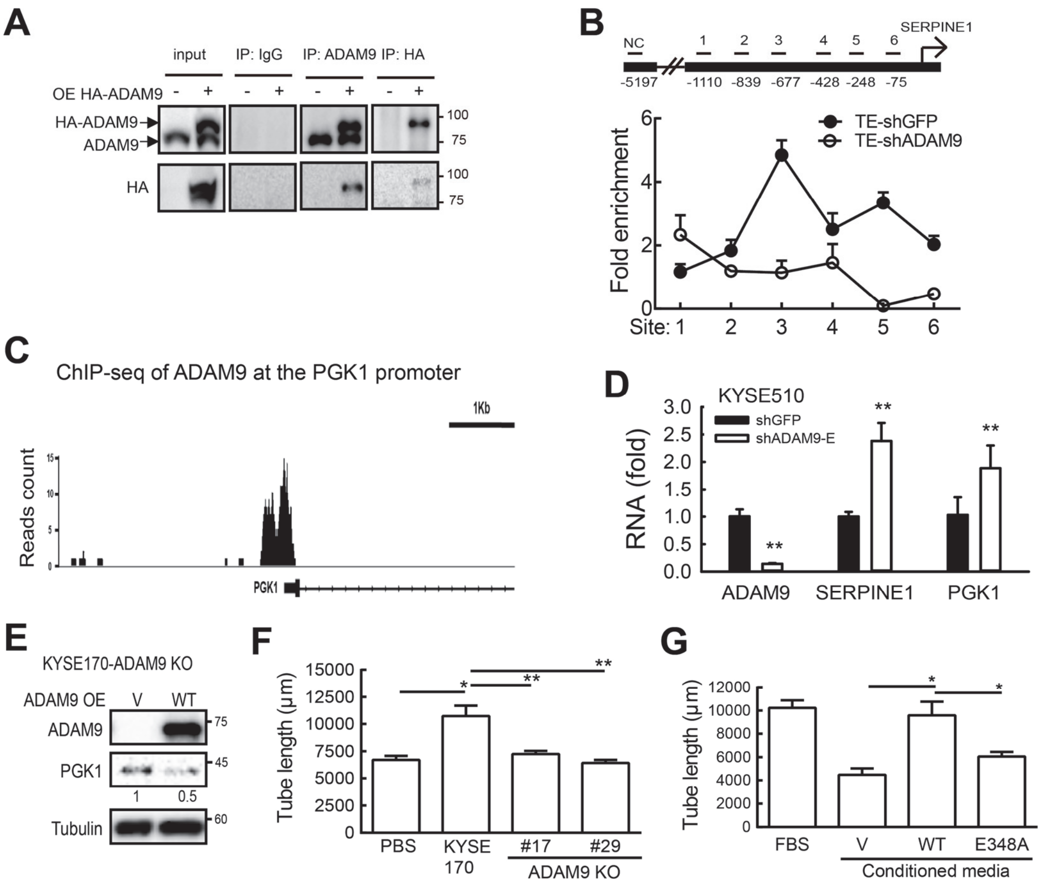

Figure S5, related to Figure 5. **(A)** Immunoprecipitation of ADAM9 in KYSE170 cells with transiently transfected HA-ADAM9 plasmids. ADAM9 proteins are immunoprecipitated from the cell lysates with antibody against ADAM9 or HA, and then detected by immunoblotting by indicated antibodies. **(B)** Enrichment of ChIP-qPCR analysis with primer sets (1 to 6) on the *SERPINE1* promoter regions in control and ADAM9 knockout TE cells using ADAM9 antibody and control IgG. **(C)** Peaks of ADAM9 occupancy are identified in *PGK1* promoter by ADAM9 ChIP-seq in KYSE170 control cells. **(D)** RT-qPCR of *ADAM9*, *SERPINE1*, and *PGK1* in control and ADAM9 knockdown KYSE510 cells. **(E)** Western blot analysis of indicated proteins in *ADAM9* KO KYSE170 cells transiently transfected with plasmids of vector (V) and ADAM9 wild type (WT) in starvation and hypoxia condition. **(F)** Tube formation assays of HUVECs treated with the conditioned media from control and *ADAM9* KO KYSE170 cells. **(G)** Tube formation assays of HUVECs treated with the conditioned media from *ADAM9* KO KYSE170 cells transiently transfected with plasmids.

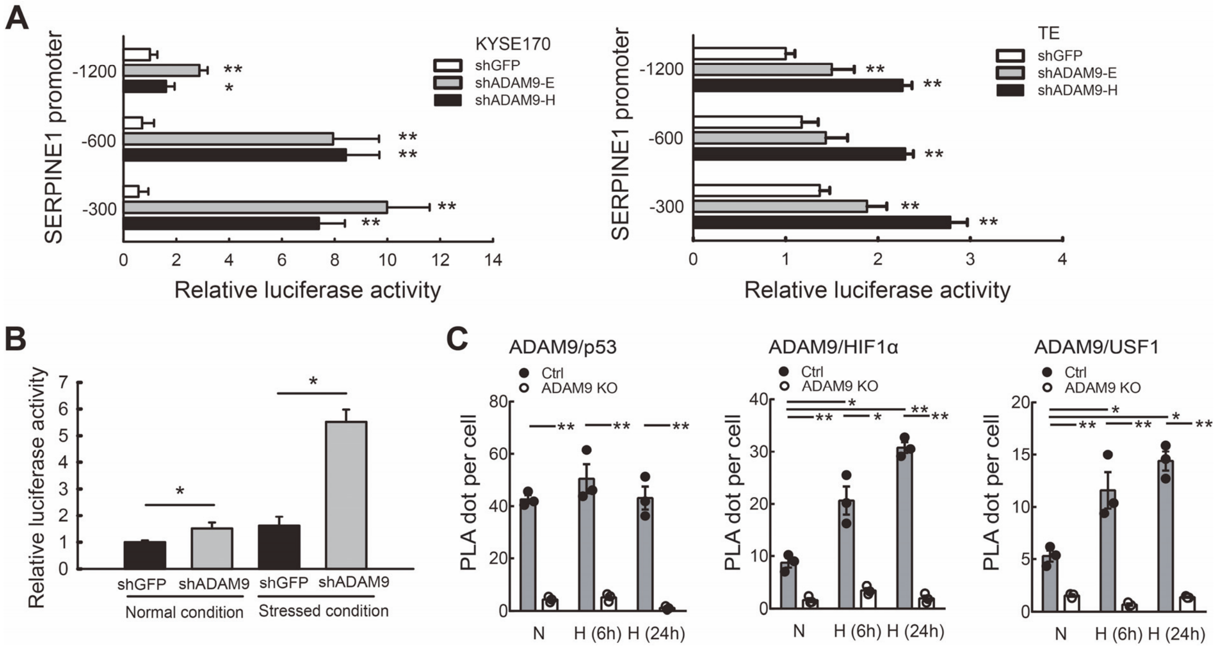

Figure S6, related to Figure 6. **(A)** The relative luciferase activity of three regions of *SERPINE1* promoter in control and ADAM9 knockdown KYSE170 and TE cells. **(B)** The relative luciferase activity in control and *ADAM9* knockdown KYSE170 cells in normal and stressed (hypoxia and starvation) culture conditions. Promoter reporter plasmids containing *SERPINE1* promoter (1200 bp) were transiently transfected into cells. The relative luciferase activity represents the dual-luciferase activity ratio (Firefly/Renilla luciferase). **(C)** PLA detection of ADAM9 and transcription factors in KYSE170 cells. Duolink PLA analysis of ADAM9/p53 complexes, ADAM9/HIF1α complexes, and ADAM9/USF1 complexes per cell. Quantification of PLA was analyzed from 3 fields.
